# An Island Grouping Genetic Algorithm for Fuzzy Partitioning Problems

**DOI:** 10.1155/2014/916371

**Published:** 2014-05-22

**Authors:** S. Salcedo-Sanz, J. Del Ser, Z. W. Geem

**Affiliations:** ^1^Department of Signal Processing and Communications, Universidad de Alcalá, 28871 Madrid, Spain; ^2^OPTIMA Area, Tecnalia Research & Innovation, 48170 Bizkaia, Spain; ^3^Department of Energy IT, Gachon University, Seongnam 461-701, Republic of Korea

## Abstract

This paper presents a novel fuzzy clustering technique based on grouping genetic algorithms (GGAs), which are a class of evolutionary algorithms especially modified to tackle grouping problems. Our approach hinges on a GGA devised for fuzzy clustering by means of a novel encoding of individuals (containing elements and clusters sections), a new fitness function (a superior modification of the Davies Bouldin index), specially tailored crossover and mutation operators, and the use of a scheme based on a local search and a parallelization process, inspired from an island-based model of evolution. The overall performance of our approach has been assessed over a number of synthetic and real fuzzy clustering problems with different objective functions and distance measures, from which it is concluded that the proposed approach shows excellent performance in all cases.

## 1. Introduction

Clustering (also known as partitioning) is an important subgroup of unsupervised learning techniques which hinges on grouping data objects into groups of* disjoint* (“crisp”)* clusters* [1–3]. A huge amount of key problems in science, engineering, and economics (e.g., bioengineering, telecommunications, energy, and risk assessment) can be formulated as clustering problems [4–8]. In this context, an important line of research related to clustering stems from the fact that, in some problems, the clusters intrinsically overlap with each other and, consequently, conventional crisp clustering algorithms are* not* suitable for dealing with this overlap [9, 10]. In these cases when an object can “partially” belong to different groups,* fuzzy clustering* algorithms have been proposed as a powerful methodology in recent years, more flexible than traditional crisp approaches and with excellent results in different real problems [11, 12].

To be specific, “fuzzy clustering” is the class of clustering problems where the boundary between clusters is ill-defined, in the sense that a given sample is allowed to belong to different clusters. As such, the notion of* fuzziness* becomes relevant since any object of the data set is assigned to a given cluster with some* membership grade*, usually set between 0 and 1 (low and high membership grade, resp.). Formally, if *X* = {**x**_1_,…, **x**_*N*_} is a set of *N* data vectors in a given observation space *S*, the goal of a fuzzy clustering algorithm is to find a partition of *X* in a finite number of *k* clusters, so that a data vector **x**_*j*_ can belong to a cluster *C*_*i*_ ∈ {*C*_1_,…, *C*_*k*_} with a degree of membership *u*_*ij*_ ∈ [0,1]. This is equivalent to finding a “partition matrix” **U** whose elements *u*_*ij*_ ∈ [0,1] (with 1 ≤ *i* ≤ *k* and 1 ≤ *j* ≤ *N*) fulfill

(1) $$\begin{array}{c} \overset{k}{\underset{i=1}{\sum }}u_{ij}=1,\;\forall j\in \left\{1,\ldots ,N\right\},\\ 0<\overset{N}{\underset{j=1}{\sum }}u_{ij}<N,\;\forall i\in \left\{1,\ldots ,k\right\}. \end{array}$$

Among the different techniques applied to fuzzy clustering that can be found in the literature, we focus on those based on the fuzzy C-means (FCM) algorithm [13], kernel methods [14, 15], statistical methods [16], clonal selection theory [17], rule-based clustering [18–20], and many different heuristic and metaheuristic approaches [21–25]. Metaheuristic algorithms have been thoroughly applied to fuzzy clustering in the last years due to their superior properties of robustness and convergence to near-optimal solutions at a moderate computational cost. Many of these approaches are based on evolutionary variants of the C-means algorithm [26, 27] or simply on direct fuzzy clustering algorithms based on genetic and evolutionary approaches [28–31], multiobjective algorithms [32], differential evolution [33], particle swarm metaheuristics [34], or evolutionary programming approaches [35].

However, despite the research activity invested on different metaheuristic approaches applied to fuzzy clustering, several avant-garde algorithms have not been explored yet in their entirety for fuzzy clustering problem. Specifically, this paper proposes a grouping genetic algorithm for fuzzy clustering. The grouping genetic algorithm (GGA) [36, 37] is a class of evolutionary algorithms whose encoding procedure is especially designed to deal with grouping problems. It has been successfully applied to a variety of problems involving grouping of items but, surprisingly, its performance has not been assessed yet when tackling fuzzy clustering problems. For this purpose, this paper builds upon preliminary work in [38] by presenting a novel grouping encoding, a modified objective function, and crossover and mutation operators specifically adapted to fuzzy clustering problems tackled via GGA heuristics. In order to further enhance the performance of the grouping genetic approach the proposed scheme also incorporates a local search stage and a parallelization of the GGA using the well-known* island* model, which can be both considered as additional novel ingredients with respect to [38]. Simulation results are presented so as to assess the performance of the proposed scheme in a number of application scenarios, based on which it is concluded that the GGA-based procedure here presented outperforms conventional fuzzy C-means methods.

The rest of this paper is structured as follows. For keeping the paper self-contained, Section 2 summarizes some key mathematical concepts to define clustering algorithms, such as different definitions of distance and objective functions.  Section 3 presents the aforementioned proposed GGA to tackle fuzzy clustering problems, along with a full description of its novel encoding, objective function, operators, local search, and parallelization approach.  Section 4 discusses the performance of the proposed approach in a variety of different synthetic and real problems. Finally, Section 5 completes the paper by discussing some concluding remarks.

## 2. Background: Fuzzy Clustering Concepts

The classification of objects into clusters aims at grouping those that are similar. The extent to which two objects are similar to each other must be quantified by using an appropriate distance measure. In this regard, Section 2.1 discusses some different definitions for distances in fuzzy clustering. The second key concept, strongly related to the first one and outlined in Section 2.2, aims at evaluating the quality of a candidate solution under test in a fuzzy clustering problem and plays a key role in the GGA described in Section 3.

### 2.1. Distances in Fuzzy Clustering

The adequate definition of the aforementioned distances plays a central role in fuzzy clustering. For instance, a norm based on Mahalanobis distance can be used with a similar definition compared to that for the crisp clustering case; namely,

(2) $$\begin{array}{c} d_{M}^{2}\left(x_{j},\mu_{i}\right)={||x_{j}-\mu_{i}||}_{\Sigma_{i}^{-1}}=\left(x_{j}-\mu_{i}\right)\cdot \Sigma^{-1}\cdot {\left(x_{j}-\mu_{i}\right)}^{T}, \end{array}$$

though, in this case, the definition of the inverse of the covariance matrix of any cluster Σ_*i*_ is slightly different and is given by

(3) $$\begin{array}{c} \Sigma_{i}^{-1}=\frac{\Sigma_{j=1}^{N}u_{ij}^{\alpha }\cdot \left(x_{j}-\mu_{i}\right)\cdot {\left(x_{j}-\mu_{i}\right)}^{T}}{\Sigma_{j=1}^{N}u_{ij}^{\alpha }}. \end{array}$$

An alternative to Mahalanobis distance, more suitable for fuzzy clustering, is the Gustafson-Kessel (GK) distance [39]. This distance metric is defined as

(4) dGK2(xj,μi)=||xi−μi|||Σ|1/dΣ−1=(xj−μi)·|Σ|1/d·Σ−1·(xj−μi)T

and allows for the consideration of elliptic clusters with different orientations. However, this distance is not able to distinguish between different cluster sizes. To circumvent this drawback, a modification of this distance was proposed in [39] in the context of the adaptive fuzzy clustering (AFC) algorithm presented therein; that is,

(5) $$\begin{array}{c} d_{\text{AFC}}^{2}\left(x_{j},\mu_{i}\right)=\left(x_{j}-\mu_{i}\right)\cdot \lambda_{i,d}\cdot \Sigma_{i}^{-1}\cdot {\left(x_{j}-\mu_{i}\right)}^{T}, \end{array}$$

where *λ*_*i*,*d*_ is a novel adaptive term associated with the smallest eigenvalue of the *i*th cluster's covariance matrix Σ_*i*_^−1^ and ***μ***_*i*_ is the centroid of those objects the centroid of those objects belonging to cluster *C*_*i*_. By using this definition, any clustering algorithm will have the chance of locating clusters with different orientation and also with different volumes.

Using a proper definition for distance plays a key role when evaluating to what extent an algorithm solves accurately the problem at hand. Exploring different functions for fuzzy clustering evaluation is thus the goal of the following section.

### 2.2. Fuzzy Clustering Evaluation

The evaluation of a given solution in a fuzzy clustering problem can be carried out using two different strategies. First, it is possible to directly evaluate the fuzzy clusters produced by the algorithm at hand by using the membership functions of the different observations of the problem. A second strategy consists of using a defuzzification process, prior to the clustering evaluation, followed by the application of any of the crisp clustering evaluation measures described below. As in the crisp clustering case, evaluation measures can be unsupervised or supervised. In the first case, direct evaluation is usually applied, whereas in the second one a defuzzification is often required, since existing labeled data are usually crisp.

#### 2.2.1. Unsupervised Evaluation

For comparison purposes with the objective (fitness) function later proposed for evaluating the performance of the algorithm, we summarize herein some of the most used unsupervised measures in the related literature.

(i) Fuzzy sum of quadratic errors (fSSE): consider
  (6) $$\begin{array}{c} \text{fSSE}\left(U\right)=\overset{k}{\underset{i=1}{\sum }}\;\underset{x\in C_{i}}{\sum }u_{ij}^{\alpha }d^{2}\left(x_{j},\mu_{i}\right), \end{array}$$
  where *α* ∈ [1, *∞*) controls the fuzziness degree of the solution; that is, values of *α* close to 1 lead the solution to a disjoint partition, whereas large values of *α* lead to more fuzzy clusters. Usually a value of *α* = 2 is selected.
(ii) Xie-Beni index (XB): defined in [40], this measure combines the sum of square errors with a term for measuring clusters separation:
  (7) $$\begin{array}{c} \text{XB}\left(U\right)=\frac{\sum_{i=1}^{k}\sum_{j=1}^{N}u_{ij}^{\alpha }\cdot d^{2}\left(x_{j},\mu_{i}\right)}{N\cdot \min_{1\leq i,j\leq k,i\neq j}\left\{d^{2}\left(\mu_{i},\mu_{j}\right)\right\}}. \end{array}$$
(iii) Fukuyama-Sugeno index (FS): the FS index [41] is similar to the XB index but, in this case, the separation between clusters is evaluated with respect to the average centroid of the *k* clusters, μ* = (1/*k*)∑_*i*=1_^*k*^ ***μ***_*i*_, instead of the centroid of the rest of clusters. Based on this rationale we obtain
  (8) $$\begin{array}{c} \text{FS}\left(U\right)=\overset{k}{\underset{i=1}{\sum }}\;\overset{N}{\underset{j=1}{\sum }}u_{ij}^{\alpha }\cdot d^{2}\left(x,\mu_{i}\right)-\overset{k}{\underset{i=1}{\sum }}\;\overset{N}{\underset{j=1}{\sum }}u_{ij}^{\alpha }\cdot d^{2}\left(\mu_{i},\mu^{*}\right). \end{array}$$

The aforementioned unsupervised measures are useful in those problems in which there is no additional information to check the quality of the generated clusters. However, there are some clustering problems in which such information is indeed available, hence allowing for supervised measures.

#### 2.2.2. Utilized Supervised Measurement: Rand Index

Among the supervised measures—sometimes called* external* measures—in this work the well-known Rand index (*R*) [42] will be utilized after defuzzification of the samples. It computes the similarity between the obtained partition and the* known* optimal solution as follows:

(9) $$\begin{array}{c} R\left(U\right)=\frac{\text{TP}+\text{FN}}{\text{TP}+\text{FP}+\text{TN}+\text{FN}}, \end{array}$$

where TP and FP are the number of correct and incorrect assignments, respectively, when the decision consists of assigning two elements to the* same* cluster; and TN and FN are the number of correct and incorrect assignments, respectively, when the decision consists of assigning two elements to* different* clusters. In other words, it is a measure of the percentage of correct decisions taken by the algorithm. Note that the value of *R* lies on the interval [0,1]: values of *R* closer to 1 indicate a better quality of the solution tested.

## 3. Proposed Grouping Genetic Algorithm for Fuzzy Clustering

As mentioned in Section 1, the grouping genetic algorithm is a class of evolutionary algorithms whose encoding strategy is especially designed to tackle grouping problems. It was first proposed by Falkenauer [36, 37], who realized that traditional genetic algorithms had difficulties when applied to grouping problems. In GGA the encoding procedure and crossover and mutation operators of traditional GAs are modified to yield a compact algorithm, with improved performance in grouping-based problems. In light of their outperforming behavior with respect to its traditional counterparts, grouping genetic algorithms have so far been successfully applied to diverse problems [43–51], including crisp clustering [52]. This paper joins the upsurge of research gravitating on GGAs by adapting this heuristic to* fuzzy* clustering problems. This section discusses several modifications we have devised towards further enhancing the performance of GGAs in fuzzy clustering, including our modifications in the encoding process, the objective function, and the crossover and mutation operators (Sections 3.1, 3.2, 3.4, and 3.5, resp.).

### 3.1. Problem Encoding

The proposed GGA for fuzzy clustering is a variable-length genetic algorithm, with a novel encoding to deal with this specific problem. The encoding is carried out by splitting each chromosome in the algorithm (or equivalently, its corresponding individual or candidate solution) into two parts: **c** = [**U** | **g**]. The first part is the* element* section composed by the partition matrix **U**, whereas the second part is denoted as the* group* section of the individual. Following this notation, a certain individual for a fuzzy clustering problem with *N* elements (objects or observations) and *k* clusters can be expressed as

(10) $$\begin{array}{c} \left[\begin{array}{c} {u_{1,1},\ldots ,u}_{1,N}\\ \vdots ,\cdots ,\vdots \\ u_{k,1},\ldots ,u_{k,N} \end{array}\;|\;g_{1},g_{2},\ldots ,g_{k}\right], \end{array}$$

where it is important to note that each element *u*_*i*,*j*_ represents the degree of membership of *j*th observation to *i*th cluster, whereas the group section keeps a list of tags associated with each of the clusters of the solution. Also observe that in this encoding, both the group and the element section have a variable length, since the number of clusters is also a variable of the problem. For the sake of clarity, let us assume the following individual:

(11) $$\begin{array}{c} \left[\begin{array}{ccccccccccccccc} 0.6 & 0.0 & 0.0 & 0.8 & 0.0 & 1.0 & 0.6 & 0.0 & 0.0 & 0.0 & 1.0 & 0.0 & 0.0 & 0.4 & 1.0\\ 0.0 & 0.0 & 1.0 & 0.2 & 0.0 & 0.0 & 0.0 & 1.0 & 0.0 & 1.0 & 0.0 & 0.0 & 0.0 & 0.2 & 0.0\\ 0.0 & 1.0 & 0.0 & 0.0 & 0.0 & 0.0 & 0.1 & 0.0 & 0.9 & 0.0 & 0.0 & 0.8 & 0.0 & 0.4 & 0.0\\ 0.4 & 0.0 & 0.0 & 0.0 & 1.0 & 0.0 & 0.3 & 0.0 & 0.1 & 0.0 & 0.0 & 0.2 & 1.0 & 0.0 & 0.0 \end{array}\;|\;1,2,3,4\right]. \end{array}$$

This chromosome encodes an individual (candidate solution) for a simple clustering problem with *N* = 15 objects: *X* = {**x**_1_,…, **x**_15_}. Note that the* group section* encodes a solution with 4 clusters, labeled “1,” “2,” “3,” and “4,” respectively. Any of the columns in the element section indicates to what extent any object **x**_*j*_ belongs to a cluster *C*_*i*_, that is, the partition matrix element *u*_*ij*_. For instance, the first column in the element section encodes a candidate fuzzy solution in which the object **x**_1_ belongs to cluster *C*_1_ with a degree of membership *u*_1,1_ = 0.6 and belongs to *C*_4_ with *u*_4,1_ = 0.4. Keeping this in mind, the aforementioned chromosome encodes an individual that represents a solution with 4 clusters, where observations *x*_2_, *x*_3_, *x*_5_, *x*_6_, *x*_8_, *x*_10_, *x*_11_, *x*_13_, and *x*_15_ belong to a single cluster, observations *x*_1_, *x*_4_, *x*_9_, and *x*_12_ belong to two different clusters with different degrees of membership, and finally observations *x*_7_ and *x*_14_ belong to three different clusters.

### 3.2. Objective Function

The proposed GGA will be run with different objective (fitness) functions to lead the search. Specifically, and for comparative purposes, we will use some of the classical objective functions for fuzzy clustering summarized in Section 2.2. In addition, in this paper we propose an adaptation of the well-known Davis-Bouldin index (used in crisp clustering problems) to the fuzzy case which, to the best of our knowledge, is novel in fuzzy clustering. We will show that the use of this modified index renders better results for the GGA than the other existing evaluation indices. The idea of the Davis-Bouldin index [53] for crisp clustering problems is to minimize the intracluster distances while simultaneously maximizing the distances among the different clusters, yielding

(12) $$\begin{array}{c} \text{DB}\left(U\right)=\frac{1}{k}\overset{k}{\underset{i=1}{\sum }}\underset{i\neq j}{\max}\left\{\frac{\sum_{x\in C_{i}}d^{2}\left(x,\mu_{i}\right)+\sum_{x\in C_{j}}d^{2}\left(x,\mu_{j}\right)}{d^{2}\left(\mu_{i},\mu_{j}\right)}\right\}. \end{array}$$

In the above expression note that small values of the conventional DB index correspond to compact and well-separated clusters. The adaptation of the DB index for fuzzy clustering proposed in this work is expressed as

(13) MDB(U,d) =1k∑i=1kmax⁡i≠j{∑t=1Nui,tαd2(xt,μi)+∑t=1Nuj,tαd2(xt,μj)d2(μi,μj)},

where *μ*_*i*_ stands for the centroid associated with cluster *C*_*i*_, calculated by considering the average of each observation weighted by the degree of membership to cluster *C*_*i*_. Note in expression (13) that the proposed MDB index explicitly depends on the particular definition considered for the distance *d*. For example, if we consider the GK distance and based on the covariance matrices of the clusters, the DB index for fuzzy clustering problems will be given by

(14) MDB(U,dGK)=1k∑i=1kmax⁡i≠j{∑t=1Nui,tαd|Σi|1/dΣi−12(xt,μi)+∑t=1Nuj,tαd|Σj|1/dΣj−12(xt,μj)min⁡{d|Σi|1/dΣi−12(μi,μj),d|Σj|1/dΣj−12(μi,μj)}}.

### 3.3. Selection Operator

In this paper we use a rank-based wheel selection mechanism, similar to the one described in [44]. First, the individuals are sorted in a list based on their quality. The position of the individuals in the list is called* rank of the individual* and is denoted as *R*_*i*_ (*i* = 1,…, *ξ*, with *ξ* standing for the number of individuals in the population of the GGA). A rank to which the best individual *x* is assigned will be *R*_*x*_ = *ξ*, whereas the second best will be *y*, *R*_*y*_ = *ξ* − 1, and so forth. A* fitness* value associated with each individual is then defined as

(15) $$\begin{array}{c} f_{i}=\frac{2\cdot R_{i}}{\xi \cdot \left(\xi +1\right)}. \end{array}$$

Note that these values are normalized between 0 and 1, depending on the position of the individual in the ranking list. It is important to note that this rank-based selection mechanism is* static*, in the sense that probabilities of survival (given by *f*_*i*_) do not depend on the generation but on the position of the individual in the list. As a toy example, consider a population formed by 5 individuals, in which individual 1 is the best quality one (*R*_1_ = 5), individual 2 is the second best (*R*_2_ = 4), and so on. In this case, the fitness associated with the individuals is {0.33,0.26,0.2,0.13,0.06}, and the associated intervals for the roulette wheel are {0–0.33,0.34–0.6,0.61–0.8,0.81–0.93,0.94–1}.

The process carried out in our algorithm consists of selecting the* parents* for crossover by using this selection mechanism. This procedure is performed* with* replacement; that is, a given individual can be selected several times as one of the parents. However, individuals in the crossover operator must be different.

### 3.4. Crossover Operator

The crossover operator implemented in the grouping genetic algorithm used in this paper is a modified version of the one initially proposed by Falkenauer in [36], but with the added bonus of being adapted to the fuzzy clustering problem. These are the main steps followed in the crossover operation.

1. Select two individuals at random and choose two crossing points in their group part.
2. Insert the elements belonging to the selected groups of the first individual into the offspring.
3. Assign the degree of membership of the inserted elements equal to the first individual.
4. Insert the elements belonging to the selected groups of the second individual into the offspring.
5. Assign the degree of membership of the inserted elements in the following way. First, the remaining degree membership after the assignment of the elements of the first individual is calculated. This remaining degree membership is then proportionally shared among the elements of the second individual.
6. Remove empty clusters, if any.
7. Modify the labels of the current groups in the offspring in order to numerate them from 1 to *k*.

A simple yet illustrative enough example follows. Let us consider two different individuals *ξ*_1_ and *ξ*_2_ that have been randomly chosen among all individuals in a given GGA population so as to perform crossover on them. The groups selected to carry out the procedure are marked in boldface:

(16) $$\begin{array}{c} \xi_{1}=\left[\begin{array}{ccccccccccccccc} 0.6 & 0.0 & 0.0 & 0.8 & 0.0 & 1.0 & 0.6 & 0.0 & 0.0 & 0.0 & 1.0 & 0.0 & 0.0 & 0.4 & 1.0\\ 0.0 & 0.0 & 1.0 & 0.2 & 0.0 & 0.0 & 0.0 & 1.0 & 0.0 & 1.0 & 0.0 & 0.0 & 0.0 & 0.2 & 0.0\\ 0.0 & 1.0 & 0.0 & 0.0 & 0.0 & 0.0 & 0.1 & 0.0 & 0.9 & 0.0 & 0.0 & 0.8 & 0.0 & 0.4 & 0.0\\ 0.4 & 0.0 & 0.0 & 0.0 & 1.0 & 0.0 & 0.3 & 0.0 & 0.1 & 0.0 & 0.0 & 0.2 & 1.0 & 0.0 & 0.0 \end{array}\;|\;1,2,3,4\right],\\ \xi_{2}=\left[\begin{array}{ccccccccccccccc} 0.8 & 0.4 & 0.0 & 0.0 & 0.0 & 0.3 & 1.0 & 0.0 & 0.0 & 0.0 & 0.0 & 0.6 & 1.0 & 0.7 & 0.0\\ 0.0 & 0.6 & 0.0 & 1.0 & 0.1 & 0.3 & 0.0 & 0.5 & 0.0 & 0.0 & 1.0 & 0.4 & 0.0 & 0.1 & 1.0\\ 0.2 & 0.0 & 1.0 & 0.0 & 0.9 & 0.4 & 0.0 & 0.5 & 0.9 & 1.0 & 0.0 & 0.0 & 0.0 & 0.2 & 0.0 \end{array}\;|\;1,2,3\right]. \end{array}$$

After steps 2 and 3 of the proposed crossover procedure (insertion of the group elements of the first individual and assignment of the degree of membership), the offspring results in

(17) $$\begin{array}{c} O=\left[\begin{array}{ccccccccccccccc} 0.0 & 0.0 & 1.0 & 0.2 & 0.0 & 0.0 & 0.0 & 1.0 & 0.0 & 1.0 & 0.0 & 0.0 & 0.0 & 0.2 & 0.0\\ 0.0 & 1.0 & 0.0 & 0.0 & 0.0 & 0.0 & 0.1 & 0.0 & 0.9 & 0.0 & 0.0 & 0.8 & 0.0 & 0.4 & 0.0 \end{array}\;|\;2,3\right]. \end{array}$$

Then the group elements of the second individual are inserted, and the membership degree is modified considering the previous existing degrees from individual 1:

(18) $$\begin{array}{c} O=\left[\begin{array}{ccccccccccccccc} 0.0 & 0.0 & 1.0 & 0.2 & 0.0 & 0.0 & 0.0 & 1.0 & 0.0 & 1.0 & 0.0 & 0.0 & 0.0 & 0.2 & 0.0\\ 0.0 & 1.0 & 0.0 & 0.0 & 0.0 & 0.0 & 0.1 & 0.0 & 0.9 & 0.0 & 0.0 & 0.8 & 0.0 & 0.4 & 0.0\\ 1.0 & 0.0 & 0.0 & 0.0 & 0.0 & 0.5 & 0.9 & 0.0 & 0.0 & 0.0 & 0.0 & 0.12 & 1.0 & 0.35 & 0.0\\ 0.0 & 0.0 & 0.0 & 0.8 & 1.0 & 0.5 & 0.0 & 0.0 & 0.1 & 0.0 & 1.0 & 0.08 & 0.0 & 0.05 & 1.0 \end{array}\;|\;{2,3,1}^{*},2^{*}\right]. \end{array}$$

There are no empty clusters. Therefore, we pass on to the final step of the crossover approach: modify the labels of current groups in the offspring in order to numerate them from 1 to *k* (4 in this case):

(19) $$\begin{array}{c} O=\left[\begin{array}{cccccccccccccccc} 0.0 & 0.0 & 1.0 & 0.2 & 0.0 & 0.0 & 0.0 & 0.0 & 1.0 & 0.0 & 1.0 & 0.0 & 0.0 & 0.0 & 0.2 & 0.0\\ 0.0 & 1.0 & 0.0 & 0.0 & 0.0 & 0.0 & 0.1 & 0.1 & 0.0 & 0.9 & 0.0 & 0.0 & 0.8 & 0.0 & 0.4 & 0.0\\ 1.0 & 0.0 & 0.0 & 0.0 & 0.0 & 0.5 & 0.9 & 0.9 & 0.0 & 0.0 & 0.0 & 0.0 & 0.12 & 1.0 & 0.35 & 0.0\\ 0.0 & 0.0 & 0.0 & 0.8 & 1.0 & 0.5 & 0.0 & 0.0 & 0.0 & 0.1 & 0.0 & 1.0 & 0.08 & 0.0 & 0.05 & 1.0 \end{array}\;|\;1,2,3,4\right]. \end{array}$$

**Figure 1:**
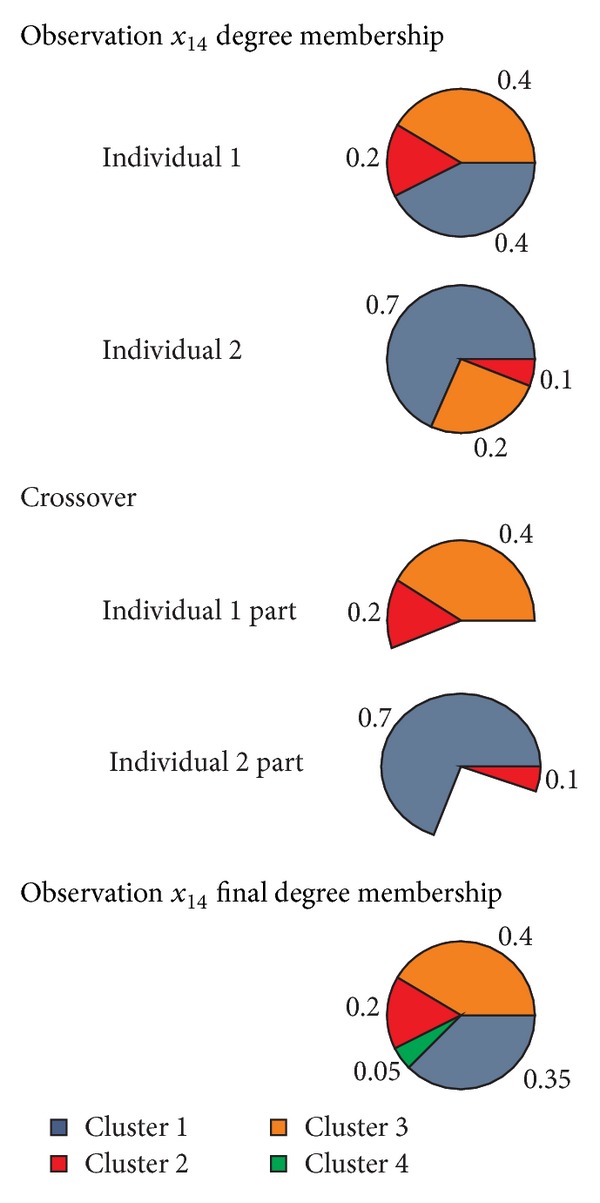
Example of the crossover operator implemented in the proposed grouping genetic algorithm for fuzzy clustering problems.

An example of the reassignment of the degree of membership in the final offspring is shown in Figure 1, where the evolution of the degrees of membership is shown for observation *x*_14_ along the crossover operation. Intuitively the crossover should be high in the first stages of the algorithm and more moderate in the last ones in order to favor the explorative behavior of the algorithm through the search space. Thus, we have implemented an adaptive crossover probability defined as

(20) $$\begin{array}{c} P_{c}\left(j\right)=P_{ci}+\frac{j}{TG}\left(P_{ci}-P_{cf}\right), \end{array}$$

where *P*_*c*_(*j*) is the crossover probability used in a given generation *j*, *TG* stands for the total number of generations of the algorithm, and *P*_*ci*_ and *P*_c*f*_ are the initial and final values of probability, respectively, which are set as inputs for the proposed algorithm.

### 3.5. Mutation Operator

Mutation operators include modifications in each individual of the population with a low probability in order to explore new regions of the search space and also to escape from local optima when the algorithm is near convergence. In this case, we have implemented two different mutation operators adapted to the fuzzy clustering problems.

(i) Mutation by* cluster splitting*: this operator consists of splitting a selected cluster into two different parts. The degrees of membership are also randomly split between the two new clusters. The samples belonging to the original cluster are assigned to the new clusters with equal probability. Note that one of the new generated clusters will keep its label in the group section of the individual, whereas the other will be assigned a new label (*k* + 1). The selection for the initial cluster to be split is carried out depending on the clusters' size, with more probability of splitting imposed on clusters of larger size. As an example, we illustrate an application of this operator in the final offspring individual of the previous example:

(21) $$\begin{array}{c} O=\left[\begin{array}{ccccccccccccccc} 0.0 & 0.0 & 1.0 & 0.2 & 0.0 & 0.0 & 0.0 & 1.0 & 0.0 & 1.0 & 0.0 & 0.0 & 0.0 & 0.2 & 0.0\\ 0.0 & 1.0 & 0.0 & 0.0 & 0.0 & 0.0 & 0.1 & 0.0 & 0.9 & 0.0 & 0.0 & 0.8 & 0.0 & 0.4 & 0.0\\ 1.0 & 0.0 & 0.0 & 0.0 & 0.0 & 0.5 & 0.9 & 0.0 & 0.0 & 0.0 & 0.0 & 0.12 & 1.0 & 0.35 & 0.0\\ 0.0 & 0.0 & 0.0 & 0.8 & 1.0 & 0.5 & 0.0 & 0.0 & 0.1 & 0.0 & 1.0 & 0.08 & 0.0 & 0.05 & 1.0 \end{array}\;|\;1,2,3,4\right]. \end{array}$$

Let us suppose that the cluster chosen to be split is cluster 1. A possible cluster splitting mutation operation would be

(22) $$\begin{array}{c} O_{m}=\left[\begin{array}{ccccccccccccccc} 0.0 & 0.0 & 0.4 & 0.08 & 0.0 & 0.0 & 0.0 & 0.4 & 0.0 & 0.4 & 0.0 & 0.0 & 0.0 & 0.08 & 0.0\\ 0.0 & 1.0 & 0.0 & 0.0 & 0.0 & 0.0 & 0.1 & 0.0 & 0.9 & 0.0 & 0.0 & 0.8 & 0.0 & 0.4 & 0.0\\ 1.0 & 0.0 & 0.0 & 0.0 & 0.0 & 0.5 & 0.9 & 0.0 & 0.0 & 0.0 & 0.0 & 0.12 & 1.0 & 0.35 & 0.0\\ 0.0 & 0.0 & 0.0 & 0.8 & 1.0 & 0.5 & 0.0 & 0.0 & 0.1 & 0.0 & 1.0 & 0.08 & 0.0 & 0.05 & 1.0\\ 0.0 & 0.0 & 0.6 & 0.12 & 0.0 & 0.0 & 0.0 & 0.6 & 0.0 & 0.6 & 0.0 & 0.0 & 0.0 & 0.12 & 0.0 \end{array}\;|\;1,2,3,4,5\right]. \end{array}$$

(ii) Mutation by* clusters merging*: this mutation consists of randomly selecting two existing clusters and merging them into just one single cluster. The degree of membership of the new cluster is the sum of the degrees of the previous ones. As in mutation by cluster splitting, the probability of choosing the clusters depends on their size. In order to illustrate this mutation, we use again the final offspring from the crossover operator example. In this case, let us consider that the selected clusters to be merged are clusters 2 and 4, resulting in

(23) $$\begin{array}{c} O_{m}=\left[\begin{array}{ccccccccccccccc} 0.0 & 0.0 & 1.0 & 0.2 & 0.0 & 0.0 & 0.0 & 1.0 & 0.0 & 1.0 & 0.0 & 0.0 & 0.0 & 0.2 & 0.0\\ 0.0 & 1.0 & 0.0 & 0.8 & 1.0 & 0.5 & 0.1 & 0.0 & 1.0 & 0.0 & 1.0 & 0.88 & 0.0 & 0.45 & 1.0\\ 1.0 & 0.0 & 0.0 & 0.0 & 0.0 & 0.5 & 0.9 & 0.0 & 0.0 & 0.0 & 0.0 & 0.12 & 1.0 & 0.35 & 0.0 \end{array}\;|\;1,2,3\right]. \end{array}$$

Analogously to the crossover operator, we also consider an adaptive version of the probability of applying the mutation operators described above. Note that we apply the two mutation operators in a serial fashion (one after the other), with independent probabilities of application. In this case, probability of mutation is made smaller in the first generations of the algorithm and larger in the last ones in order to have more opportunities to escape from local minima in the last stages of the evolutionary process; that is,

(24) $$\begin{array}{c} P_{m}\left(j\right)=P_{mi}+\frac{j}{TG}\left(P_{mf}-P_{mi}\right), \end{array}$$

where *P*_*m*_(*j*) is the probability of mutation used in a given generation *j*, *TG* stands for the total number of generations of the algorithm, and *P*_*mf*_ and *P*_*mi*_ are the final and initial values of probability considered, respectively.

### 3.6. Replacement and Elitism

In the proposed GGA, the population at a given generation *j* + 1 is obtained by replacement of the individuals in the population at generation *j*, through the application of the selection, crossover, and mutation operators described above. An elitist scheme is also applied: the best individual in generation *j* is automatically passed on to the population of generation *j* + 1, ensuring that the best solution encountered so far in the evolution is always kept by the algorithm.

### 3.7. Local Search

We use a local search procedure to try to find local optima in a close neighborhood of a given individual. The proposed local search is based on minor modifications of the current individual, as far as they produce an increase of the associated objective function: the local search changes the degree of membership of the observations, starting by one randomly chosen. The changes in the degree of membership are randomly generated. We finally keep the assignment with the largest objective function. Since this local search procedure is a time-consuming operation, it is applied to a given individual with a small probability, *p*_*b*_, that is modified between an initial and final value in the algorithm in the same way that the crossover probability is modified.

### 3.8. An Island Model to Improve the Algorithm's Performance

In order to improve the performance of the proposed GGA, an island model is considered for its parallelization. In this context, *S* subpopulations (islands) are set in such a way that the evolution in each island is forced to be independent but the migration of good individuals is allowed between islands. We consider an elitist migration model, in which only the best individual in each island migrates and substitutes a randomly chosen individual in one of the other islands. There is a probability of migration *p*_*e*_ predefined in the algorithm. The migration process is summarized in the following steps.

1. Choose the best individual in each island.
2. Randomly choose the island toward which each individual will migrate.
3. Randomly choose an individual in the destiny island and change it by the migrating individual.

## 4. Experiments and Results

This section summarizes and discusses the experimental work we have carried out in order to assess the performance of our proposed GGA approach. We have explored a number of variations of the proposed GGA (by combining different distances and/or objective functions) in a variety of fuzzy clustering scenarios (which, as will be shown later, exhibit an increasing degree of complexity).  Table 1 lists the values of the GGA parameters used in all the simulations carried out in this paper. These values have been found to be the most appropriate after a number of side experiments, not shown for the sake of brevity. The algorithm presented here is compared with the fuzzy C-means (FCM) [13] algorithm because it has been successfully applied to many real clustering problems and applications characterized by different levels of complexity [26, 27].

For reasons made clearer in what follows, the experimental setup for comparing the considered algorithms will be divided into two different parts, characterized by using synthetic and real data (Sections 4.1 and 4.2, resp.).

### 4.1. Synthetic Data

#### 4.1.1. Experiment 1 with Synthetic Data: Spherical Clusters

In this first experiment, we test the performance of the proposed GGA in a two-dimensional clustering problem, defined by 300 observations randomly generated using a Gaussian distribution from 8 equiprobable classes, with mean values *μ*_1_ = (−1,1), *μ*_2_ = (2, −2), *μ*_3_ = (1,0), *μ*_4_ = (3, −1), *μ*_5_ = (−1, −1), *μ*_6_ = (−1, −3), *μ*_7_ = (1,2), and *μ*_8_ = (3,1) and covariance matrices:

(25) $$\begin{array}{c} \Sigma_{1}=\Sigma_{2}=\cdots =\Sigma_{8}=\left[\begin{array}{cc} 0.35^{2} & 0\\ 0 & 0.35^{2} \end{array}\right]. \end{array}$$

**Figure 2:**
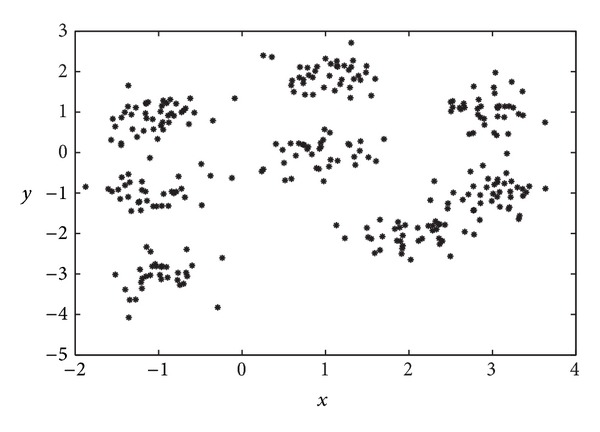
Two-dimensional representation of data for the first synthetic clustering example (spherical data). See the main text for further details.

Note that this procedure results in a problem characterized by spherical clusters.  Figure 2 illustrates the two-dimensional distribution of the observations following the above statistical distribution.

We have applied to this problem a number of configurations of the proposed GGA—with MDB, XB, and FS objective (fitness) functions—and the FCM algorithm fed with the real number of clusters as a priori information. At this point it is important to emphasize that the proposed GGA is able to infer the number of clusters within the problem, whereas the FCM requires this parameter to be set before execution (namely, *C* in the above description of FCM). To circumvent this issue, side simulations have been run for FCM and the considered scenario by varying *C* over a wide range of integer values, from which the value rendering the best metric value has been selected for comparison. Also included is the GGA approach from [38] in order to assess the impact of the novel aspects of the island-based GGA proposed here.

**Figure 3:**
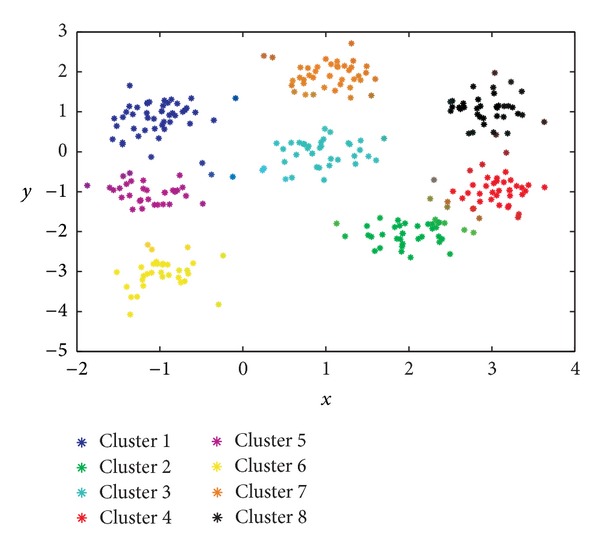
Representation of the best result obtained by the proposed GGA with MDB fitness function in the first synthetic clustering example.

**Figure 4:**
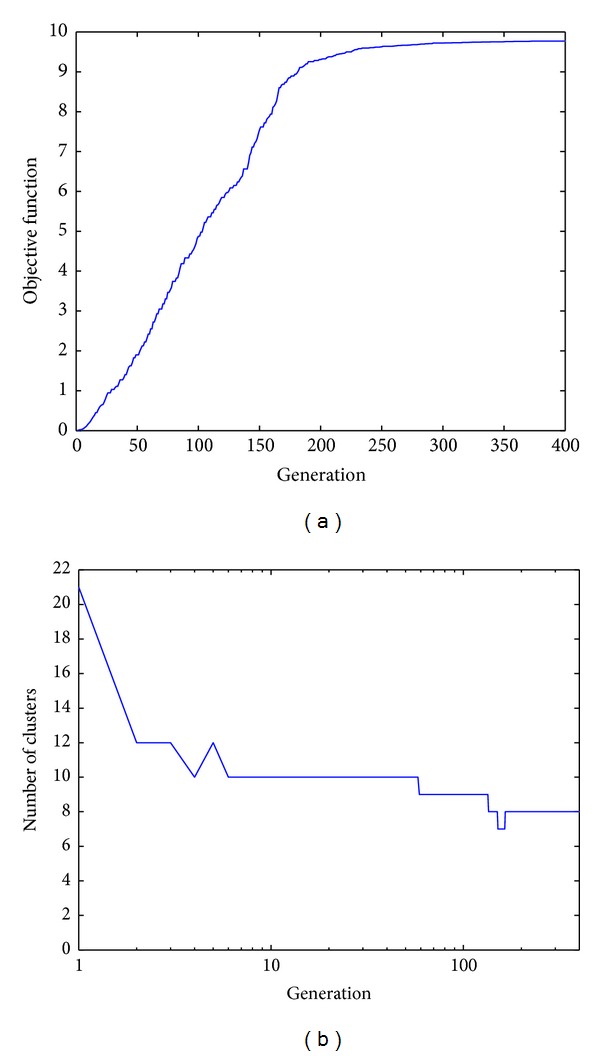
Evolution, as a function of the number of generations involved, of (a) the objective (fitness) function (MDB) and (b) the number of clusters obtained.

**Figure 5:**
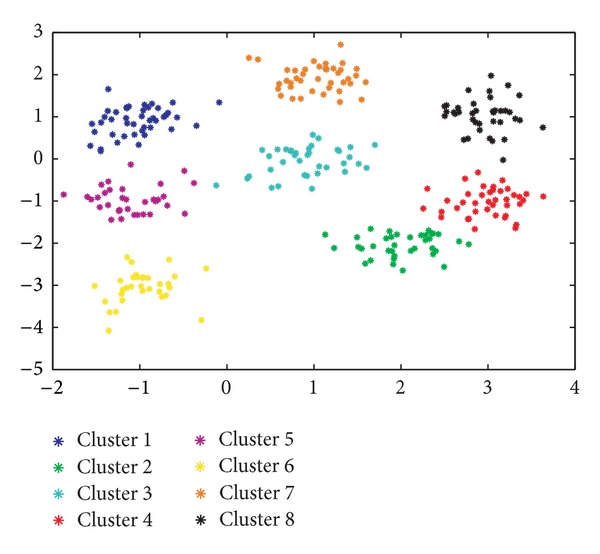
Final solution obtained by the proposed GGA (MDB index) after the defuzzification process in the first synthetic clustering problem considered.

Having said this, Table 2 lists the supervised evaluation of the results obtained by the aforementioned algorithms. Note that the proposed GGA with the three different objective functions obtains better results than the FCM algorithm. In particular, our GGA with the MDB index exhibits the best behavior (*R* = 0.9937), higher than that of the conventional FCM algorithm (*R* = 0.9712) and the GGA with MDB index from [38] (*R* = 0.9918). In addition, note that the GGA with MDB and XB indexes achieves the solution with the optimal number of clusters (i.e., 8). In order to better describe the behavior of the best algorithm (the GGA with our MBD index), it would be very interesting to have a closer look at Figures 3 to 5.

- Figure 3 represents the two-dimensional distributions of the 8 clusters found. The color of each observation has been obtained as a combination of those colors representing each cluster, weighted by the degree of membership of each observation.
- Figures 4(a) and 4(b) depict, as a function of the number of generations considered, the evolution of the objective function and that of the number of clusters, respectively, in what is the best solution found for this problem. It is worth noting that the algorithm is able to isolate the 8 clusters of the data set with a value of the objective function of 9.7688.
- Finally, Figure 5 shows the final solution after the defuzzification process, illustrating the ability of the proposed algorithm to find the 8 clusters.

The question arising from this first experiment lies on how the proposed fuzzy clustering approach works when facing clusters that are not spherical or exhibiting different distributions. This is the rationale behind the following second synthetic experiment.

#### 4.1.2. Experiment 2 with Synthetic Data: Unbalanced Data

We now test the performance of the proposed GGA in a different two-dimensional clustering problem, defined by 400 randomly generated objects following a distribution drawn from 3 Gaussian classes with probabilities *p*_1_ = 0.5, *p*_2_ = 0.33, and *p*_3_ = 0.17. The mean values of each of such classes are *μ*_1_ = (0,2), *μ*_2_ = (−1, −1), and *μ*_3_ = (2, −1), whereas their covariance matrices are given by

(26) Σ1=[12000.82],Σ2=[0.62000.42],Σ3=[0.32000.52].

**Figure 6:**
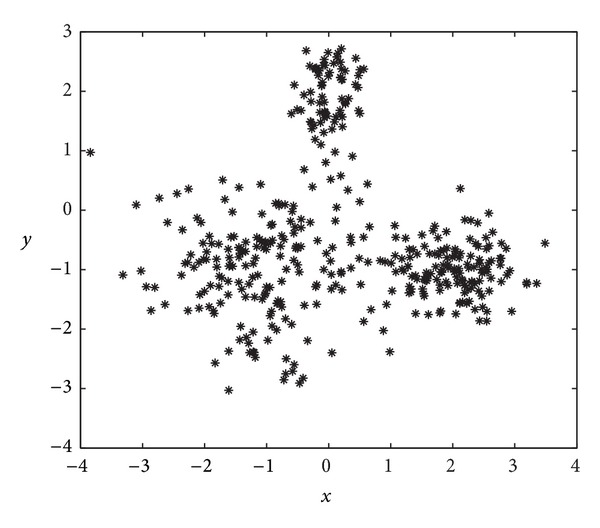
Data for the second synthetic clustering example (unbalanced data). See the main text for further details.

Note that, in this case, the classes are not spherical and have different distributions.  Figure 6 displays the observations generated for this instance.

**Figure 7:**
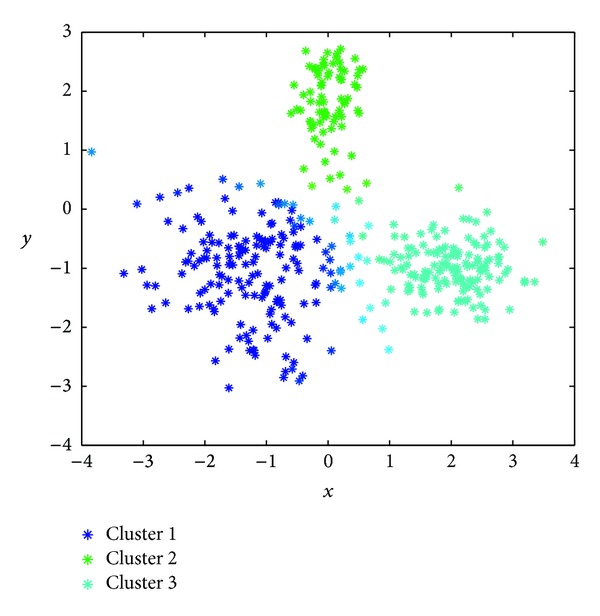
Best result obtained by the proposed GGA (MDB index) in the second synthetic clustering example.

Table 3 shows, in terms of the Rand index, the results obtained by the proposed GGA with MDB, XB, and FS indexes and the previous scheme from [38] with the same set of indexes and those achieved by the FCM algorithm. As shown in this table, the GGA with MDB and XB indexes obtains similar results (better than the FCM), whereas the result of the GGA with FS index is slightly worse than the result of the FCM algorithm. The best results correspond to the here proposed GGA algorithm with the MDB index, rendering a value of *R* = 0.9284 (higher than that of the GGA approach from [38] with the same index and the FCM algorithm) and, what is very important, finding the 3 clusters hidden in the data. Finally, Figure 7 illustrates, in a more intuitive way, the fuzzy clustering reached by the proposed GGA using the MDB index as objective function.

#### 4.1.3. Experiment 3 with Synthetic Data: Heterogeneous Clusters

The goal of this final synthetic experiment consists of exploring the effects of using different distances in the MDB objective function rendering the best results obtained by the proposed GGA. We again set up another two-dimensional clustering problem defined by 300 Gaussian-distributed objects, but in this case the Gaussian distribution is randomly drawn from 6 classes with probabilities *p*_1_ = 0.1, *p*_2_ = 0.1, *p*_3_ = 0.1, *p*_4_ = 0.25, *p*_5_ = 0.25, and *p*_6_ = 0.2. Means of the classes are set as *μ*_1_ = (−2, −2), *μ*_2_ = (0, −2), *μ*_3_ = (2, −2), *μ*_4_ = (0,0.5), *μ*_5_ = (−1.5,2), and *μ*_6_ = (2,2.5), whereas the covariance matrices are selected to be

(27) Σ1=[0.30.280.280.3],Σ2=[0.02000.02],Σ3=[0.3−0.28−0.280.3],Σ4=[0.46000.46],Σ5=[0.2000.2],Σ6=[0.5000.5].

**Figure 8:**
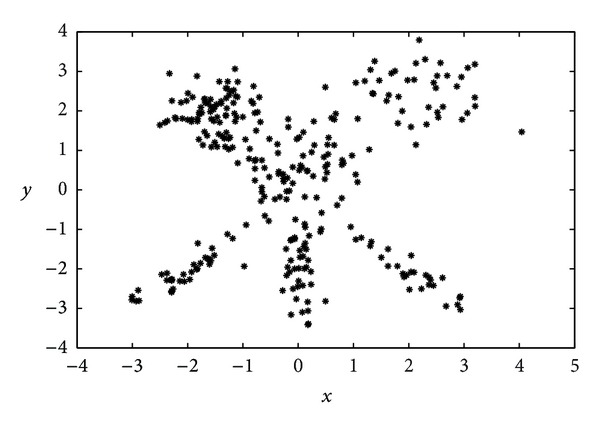
Data for the third synthetic clustering example (heterogeneous data).

For illustrative purposes, Figure 8 displays the observations generated for this instance.

**Figure 9:**
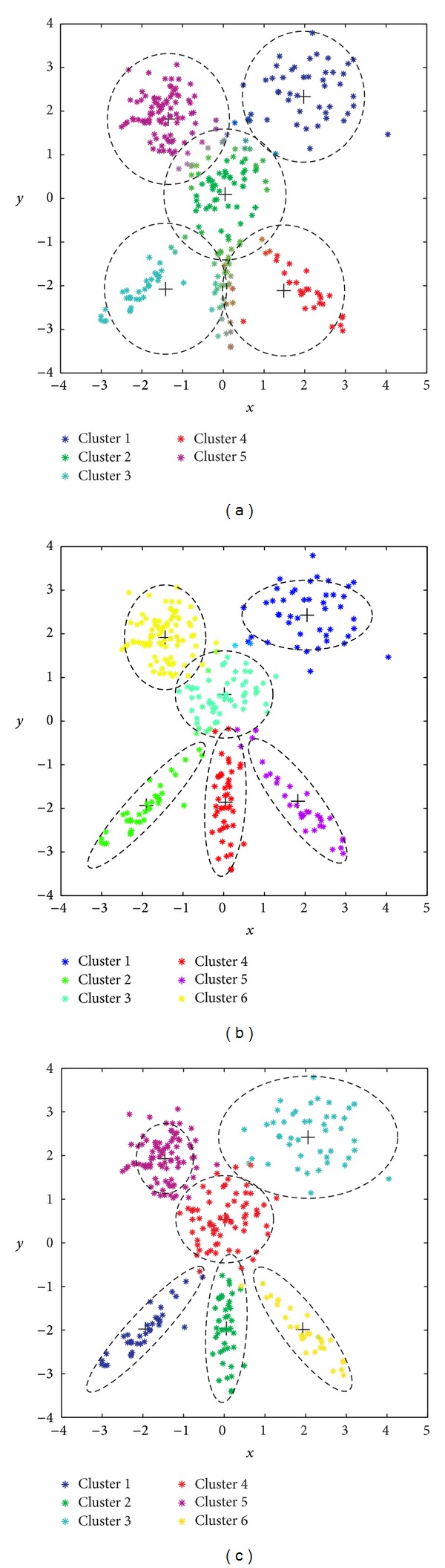
Best solutions found by the GGA with MDB index and different distances: (a) Euclidean distance; (b) GK distance; (c) AFC distance.

The analysis we have carried out in this case consists of comparing the GGA with the MDB index as objective function (which has obtained the best results in previous experiments), but using* different* distance within MDB metrics. Specifically, we will show the effect of including Euclidean, GK, and AFC distances within the proposed GGA.  Figure 9(a) represents the solution found by the GGA with MDB index and Euclidean distance. Note that the algorithm is not able to distinguish* nonspheric clusters*. By contrast, Figure 9(b) shows the result obtained by the proposed GGA with the MDB index and the GK distance. In this case, the algorithm is able to detect the structure of the problem, as can be checked out in the detection of the elliptic clusters at the bottom of the figure. Finally, Figure 9(c) shows the result obtained by the proposed GGA with MDB index and the AFC distance. Note that in this case the adaptive distance measure allows detecting clusters of* different sizes*, as the large ones at the topmost part of the figure.

The analysis of the GGA performance in this problem proceeds by comparing the results obtained in terms of the Rand index (supervised measure).  Table 4 lists the results computed by the proposed GGA, with MDB index and the different distances considered, compared to the results achieved by the FCM approach (with Euclidean distance, which has been found to be the best for the FCM algorithm). Note that the strategy using the proposed GGA with our MDB fitness function and the AFC distance exhibits the best performance, not only because it reaches the highest Rand index (*R* = 0.9670), but also because it properly detects the 6 clusters hidden in the data. Furthermore, only the proposed GGA approach with MDB index and AFC and GK distances is able to locate the correct number of clusters in the final solution.

### 4.2. Real Data

#### 4.2.1. Real Problem 1: Character Recognition

This problem can be stated as follows: let *I* be a character, a two-dimensional image, in which each pixel, *I*_*ij*_, has been converted to black and white, with black pixels forming the character image. The goal is to optimally segment all the black pixels into clusters, in such a way that a final step of comparison with a reference set can be carried out, with the aim of recognizing the character of the image.

**Figure 10:**
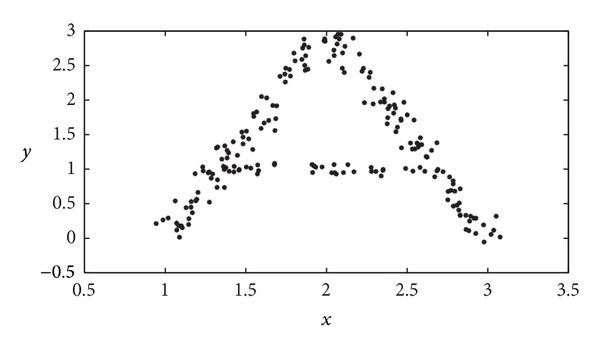
Data for the character recognition problem considered.

**Figure 11:**
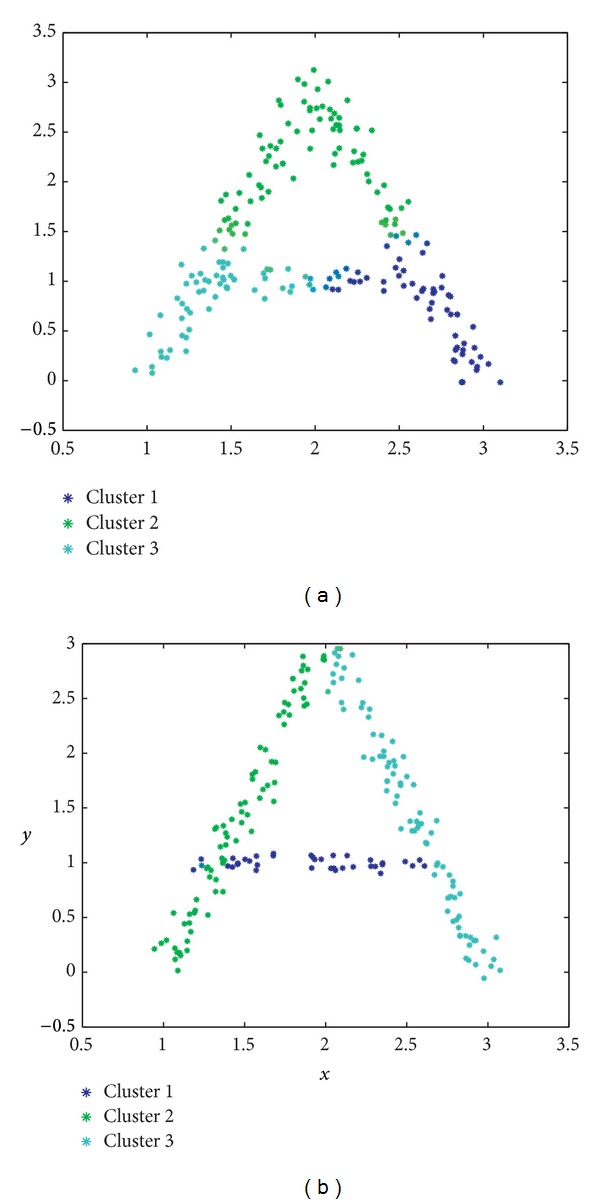
Results obtained in the character recognition problem by the proposed GGA: (a) Euclidean distance; (b) GK distance.

To illustrate the feasibility of our procedure, we have made use of an example, given by the character “*A*” depicted by means of the different samples in the image represented in Figure 10. The performance of the proposed GGA in the recognition of this character is given in Figures 11(a) and 11(b), which display the results achieved by the GGA using our MDB index as objective function and the Euclidean and GK distances, respectively. It is important to note how the proposed GGA approach using the GK distance is able to correctly allocate the three segments that form the *A* character. The GGA with the Euclidean distance does not provide, however, as good results as those depicted in Figure 11(a). To further assess the feasibility of our proposal, Table 5 summarizes a quantitative comparison in terms of the Rand index. The GGA with MDB index and GK distance is the best among all the algorithms compared, whereas the GGA using the MDB index and either Euclidean or AFC distances obtains similar results to those of the FCM approach. The approach that leads to the best solution of this problem is the proposed GGA by using our MDB fitness function along with the GK distance: it is able to correctly find the 3 segments (clusters of points) with the highest Rand index (*R* = 0.9380).

#### 4.2.2. Real Problem 2: Diabetes Data Set

The data set called “diabetes” (UCI machine learning repository, see [54]) is a well-known problem in classification and clustering involving the diagnosis of diabetes patients, as defined by the World Health Organization. This data base is formed by 768 data vectors, containing, in turn, 8 features that represent medical conditions of the patients, such as age, arterial pressure, or body mass index. The observations belong to two classes, 500 of which belong to a negative diabetes diagnosis and 268 to a positive one. The results obtained by the proposed GGA assisted by the MDB index (which has been found to be the best) are shown in Table 6, in terms of percentage of correct classification. Note that the GGA-MDB with GK distance is the best algorithm among all compared, with a percentage of correct classification over 83%.

## 5. Conclusions

In this paper we have presented a grouping genetic algorithm for fuzzy clustering problems. The main contributions of this work are (1) a novel encoding approach of the individuals involved in the evolutionary process, containing information not only of the partition matrix elements, but also of the clusters being obtained; (2) a novel fitness function based on a modification of the Davis-Bouldin index for its efficient use in fuzzy clustering problems and that enables the chance of introducing norms adapted to any problem; (3) novel crossover and mutation operators particularly derived to achieve the effective evolution of the individuals; and (4) a local search and parallelization-based scheme of the algorithm aimed at improving its overall performance.

Indeed, such performance has been explored in a variety of experiments, both synthetically generated and based on practical problems. The experimental work devised—based on different fuzzy problems characterized by an increasing degree of complexity (clusters with different distribution, volume, and orientation)—proves that our algorithm (using our proposed fitness function with distances such as the Gustafson-Kessel distance or the one established for the adaptive fuzzy clustering) exhibits a significantly better performance than that achieved by the fuzzy C-means algorithm.

## Figures and Tables

**Table 1:** GGA parameters values used in the experiments of the paper.

Parameter	Meaning	Value
Ps	Population size	20
*S*	Number of subpopulations	4
TG	Maximum number of generations	400
*P*_*ci*_	Initial crossover probability	0.8
*P*_*cf*_	Final crossover probability	0.6
*P*_*mi*_	Initial mutation probability	0.05
*P*_*mf*_	Final crossover probability	0.1
*P*_*bi*_	Initial local search probability	0.1
*P*_*bf*_	Final local search probability	0.05
*P*_*e*_	Probability of migrating (islands model)	0.03
*α*	Fuzziness degree	2

**Table 2:** Comparison of the results (in terms of the number of clusters finally found and as a function of the Rand index) obtained by the proposed GGA algorithm with MDB, XB, and FS indexes, respectively, with the previous GGA in [38] and the FCM algorithm in the first synthetic clustering problem considered.

Algorithm	Number of clusters	Rand index
Proposed GGA (MDB index)	8	** 0.9937**
Proposed GGA (XB index)	8	0.9805
Proposed GGA (FS index)	9	0.9874
GGA from [38] (MDB index)	8	0.9918
GGA from [38] (XB index)	8	0.9785
GGA from [38] (FS index)	9	0.9847
FCM	8	0.9712

**Table 3:** Comparison of the results (in terms of the number of clusters finally found and as a function of the Rand index) obtained by the proposed GGA algorithm with MDB, XB, and FS indexes, respectively, with the previous GGA in [38] and the FCM algorithm in the second synthetic clustering problem considered.

Algorithm	Number of clusters	Rand index
Proposed GGA (MDB index)	3	** 0.9284**
Proposed GGA (XB index)	3	0.9203
Proposed GGA (FS index)	7	0.7998
GGA from [38] (MDB index)	3	0.9177
GGA from [38] (XB index)	3	0.9128
GGA from [38] (FS index)	7	0.7606
FCM	4	0.8561

**Table 4:** Comparison of the results (in terms of the number of clusters finally found and as a function of the Rand index) obtained by the proposed GGA algorithm with MDB index and different distances and the FCM algorithm (with Euclidean distance, which has been found to be the best for this algorithm) in the third synthetic clustering problem considered. See the main text for further details.

Algorithm	Distance	Number of clusters	Rand index
GGA (MDB index)	Euclidean	5	0.8989
GGA (MDB index)	GK	6	0.9475
GGA (MDB index)	AFC	6	0.9670
FCM	Euclidean	6	0.9416

**Table 5:** Comparison of the results obtained by the proposed GGA algorithm with MDB index and different distances and the FCM algorithm in the character recognition problem.

Algorithm	Distance	Number of clusters	Rand index
GGA (MDB index)	Euclidean	5	0.6606
GGA (MDB index)	GK	3	0.9380
GGA (MDB index)	AFC	3	0.6906
FCM	Euclidean	5	0.6781

**Table 6:** Comparison of the results obtained by the proposed GGA algorithm with MDB index and different distances and the FCM algorithm in the diabetes problem. *P*_*C*_ (%) stands for the probability of correct classification.

Algorithm	Distance	*P*_*C*_ (%)
GGA (MDB index)	Euclidean	0.7246
GGA (MDB index)	GK	0.8348
GGA (MDB index)	AFC	0.7406
FCM	Euclidean	0.6601
